# Extensively Drug-Resistant *Acinetobacter baumannii*

**DOI:** 10.3201/eid1506.081006

**Published:** 2009-06

**Authors:** Yohei Doi, Shahid Husain, Brian A. Potoski, Kenneth R. McCurry, David L. Paterson

**Affiliations:** University of Pittsburgh Medical Center, Pittsburgh, Pennsylvania, USA (Y. Doi, B.A. Potoski, D.L. Paterson); University of Toronto, Toronto, Ontario, Canada (S. Husain); Toronto General Hospital, Toronto (S. Husain); Cleveland Clinic, Cleveland, Ohio, USA (K.R. McCurry); University of Queensland Centre for Clinical Research, Brisbane, Queensland, Australia (D.L. Paterson); Royal Brisbane and Women’s Hospital, Brisbane (D.L. Paterson)

**Keywords:** Acinetobacter baumannii, bacteria, antimicrobial resistance, extensively drug resistant, colistin, Pennsylvania, USA, letter

**To the Editor:** In the 1990s, patients infected with vancomycin-resistant *Enterococcus faecium* were successfully treated with new antimicrobial drugs. However, it is unlikely that new antimicrobial drugs will be available in the near future to treat patients infected with gram-negative pathogens such as *Acinetobacter baumannii* (*1*). No new antimicrobial drugs active against this organism are currently in clinical trials (www.clinicaltrials.gov). We report a patient infected with *A*. *baumannii* that lacked susceptibility to all commercially available antimicrobial drugs.

The patient, a 55-year-old woman, had a prolonged stay in an intensive care unit at the University of Pittsburgh Medical Center (Pittsburgh, PA, USA) after undergoing lung transplantation. In the tenth postoperative week, ventilator-associated pneumonia developed, which was caused by *A*. *baumanni* that lacked susceptibility to all antimicrobial drugs tested except colistin (MIC 0.5 μg/mL). Therapy with colistin and tigecycline was begun. Colistin was administered intravenously and by inhalation. Although the pneumonia showed radiographic response to the antimicrobial drug therapy, *A*. *baumannii* continued to be isolated from respiratory secretions on numerous occasions. Despite another course of therapy with colistin and cefepime, the patient never recovered from respiratory failure. She eventually died of sepsis caused by vancomycin-resistant *E*. *faecium*. An *A*. *baumannii* isolate obtained just before she died lacked susceptibility to all commercially available antimicrobial drugs (Table).

**Table:** MICs and antimicrobial drug susceptibility for an extensively drug-resistant strain of Acinetobacter baumannii*

Drugs	MIC, μg/mL	Interpretation
Carbapanems		
Imipenem	>32	Resistant
Meropenem	>32	Resistant
Penicillins		
Ampicillin/sulbactam	32	Resistant
Piperacillin/tazobactam	>256	Resistant
Cephalosporins		
Ceftazidime	48	Resistant
Cefepime	16	Intermediate
Aminoglycosides		
Gentamicin	>256	Resistant
Tobramycin	>256	Resistant
Amikacin	>256	Resistant
Others		
Ciprofloxacin	>32	Resistant
Tigecycline	2	Intermediate
Colistin	>1,024	Resistant

*Susceptibility testing was performed by using the Etest (AB Biodisk, Solna, Sweden), except for colistin, for which the standard agar dilution method was used. Interpretation was according to breakpoints provided by the Clinical and Laboratory Standards Institute (CLSI; Wayne, PA, USA). No tigecycline breakpoints for *Acinetobacter* spp. are provided by the CLSI, the European Committee on Antimicrobial Susceptibility Testing (Basel, Switzerland), or the US Food and Drug Administration (Silver Spring, MD, USA). Breakpoints of the British Society for Antimicrobial Chemotherapy (Birmingham, UK) are indicated for tigecycline.

Multidrug-resistant *A*. *baumannii* has emerged as a substantial problem worldwide (*2*). Such strains are typically resistant to all β-lactams and fluoroquinolones and require salvage therapy with colistin, amikacin, or tigecycline. Unfortunately, notably high-level resistance to colistin and amikacin was found in the isolate we have described (Table). Tigecycline, a newly available glycylcycline antimicrobial drug, showed intermediate susceptibility. No randomized trials have been performed to specifically evaluate combination antimicrobial drug therapy for treatment of infection with *A*. *baumannii*.

Considerable media attention has been paid to extensively drug-resistant (XDR) strains of *Mycobacterium tuberculosis* (*3*). Infections with XDR strains are extremely difficult to treat and pose considerable infection control issues. We recently proposed that gram-negative bacilli lacking susceptibility to all commercially available antimicrobial drugs also be referred to as XDR because no therapeutic options are available (*4*).

Numerous outbreaks of *A*. *baumannii* infection have been reported worldwide (*5*). Unfortunately, multidrug-resistant *A*. *baumannii* strains have become endemic in some institutions. Experimental and clinical isolates lacking susceptibility to colistin, often considered the drug of last resort, are increasingly being reported (*6*–*8*). Therefore, we alert healthcare workers to the need for stringent care in adhering to infection control precautions when caring for patients infected with XDR *A*. *baumannii*. Use of contact isolation precautions, enhanced environmental cleaning, removal of sources of infection from the hospital environment, and prudent use of antimicrobial drugs can contribute to control of such outbreaks (*5*). Fortunately, no spread of the XDR strain affecting this patient occurred. A crisis is looming should XDR *A*. *baumannii* become established pathogens in hospitals.
